# Can Diffusion-Weighted Imaging Serve as an Imaging Biomarker for Acute Bacterial Rhinosinusitis?

**DOI:** 10.7759/cureus.9893

**Published:** 2020-08-20

**Authors:** Alok A Bhatt, Angela M Donaldson, Osarenoma U Olomu, Vivek Gupta, Sukhwinder Johnny S Sandhu

**Affiliations:** 1 Radiology, Mayo Clinic, Jacksonville, USA; 2 Otorhinolaryngology, Mayo Clinic, Jacksonville, USA

**Keywords:** mri, dwi, acute sinusitis, acute bacterial rhinosinusitis, anti-microbial therapy, imaging biomarker

## Abstract

Acute rhinosinusitis is defined as symptomatic inflammation of the mucosal lining of the nasal cavity and paranasal sinuses lasting less than four weeks. It is most commonly secondary to viral infection but is often challenging to distinguish from bacterial etiologies. Even with recommendations from several specialty societies, there continues to be a frequent practice of overprescribing oral antibiotics for acute rhinosinusitis, thus leading to multidrug-resistant organisms, and rendering oral medication useless when actually clinically warranted. We observed a potential non-invasive imaging biomarker that could predict which patients would benefit from anti-microbial therapy.

Often computed tomography (CT) imaging is obtained by the provider before consultation with the otolaryngologist, sometimes leading to unnecessary radiation to the patient. In addition, there are no clear CT findings to make the diagnosis of acute rhinosinusitis. The diagnosis is challenging for all clinicians involved, and therefore, additional signs on other imaging modalities would be helpful. We present a series of four patients with incidentally discovered culture-positive acute rhinosinusitis.

Patients with incidentally discovered culture-positive acute rhinosinusitis were found to also have magnetic resonance imaging (MRI) that showed corresponding restricted diffusion on diffusion-weighted imaging (DWI).

An imaging biomarker for acute bacterial rhinosinusitis may improve the appropriate use of antibiotic therapy. DWI MRI should be further investigated as a potential candidate screening modality.

## Introduction

Acute sinusitis accounts for over 400,000 emergency department visits a year [1]. Sinusitis affects nearly one in every eight adults in the United States with nearly 30 million diagnosed cases a year. It is responsible for 11 billion dollars in direct costs per year without consideration to loss of productivity and quality of life. Nearly 20% of all antibiotic prescriptions are for sinusitis [2]. Ultimately, 90% of these prescriptions are deemed not indicated resulting in multidrug resistance, unnecessary costs, and patient side effects. One study by Smith et al. estimated that more than 80% of patients who present acutely with nasal congestion and discharge will receive oral antibiotics, despite the fact that less than 2% of acute rhinosinusitis cases are bacterial in etiology [3].

The most recent clinical practice guidelines consensus statement published in 2015 defines acute rhinosinusitis as less than four weeks of purulent nasal drainage and nasal obstruction, facial pain/pressure, or both that persist without improvement for at least 10 days or if symptoms worsen after initial improvement [2,4]. The current recommendation from the American College of Radiology (ACR) Appropriateness Criteria and American Academy of Otolaryngology-Head and Neck Surgery is that diagnosis of acute sinusitis should be based on clinical evaluation and history [2,4,5]. Despite these guidelines, general practice includes imaging of patients with computed tomography (CT), exposing them to unnecessary radiation, and often still without a definitive diagnosis. This is especially problematic in the emergency setting where there is pressure to diagnose and treat patients accurately and efficiently, sometimes based solely on imaging.

Since the diagnosis of acute bacterial sinusitis is difficult with both clinical presentation and sinus CT, the use of other imaging modalities should be explored. In our experience, we have noticed that acute bacterial sinusitis can occasionally have restricted diffusion on magnetic resonance imaging (MRI).

## Case presentation

We identified four patients who had incidentally discovered culture-positive acute bacterial rhinosinusitis on MRI. In these cases, we observed restricted diffusion on diffusion-weighted imaging (DWI). The four patients ranged in age from eight to 59 years. There were three males and one female, none were immune-compromised, and all four were referred to MRI for reasons not referable to the sinuses. They had imaging for reasons such as headache, post-treatment surveillance, or pre-operative planning.

**Figure 1:**
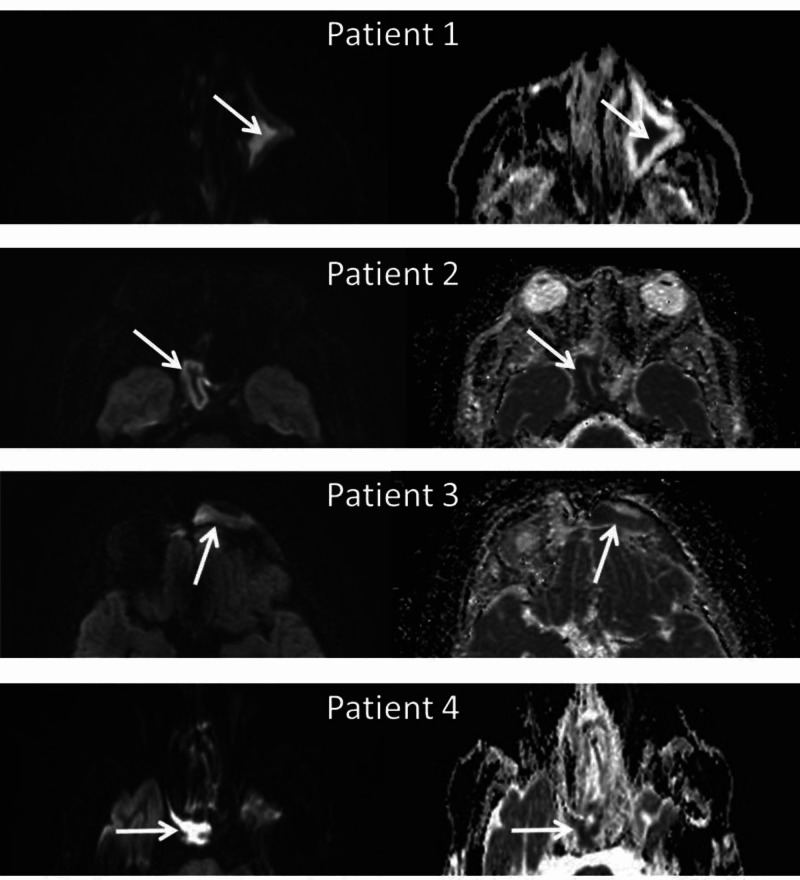
Diffusion-weighted imaging (DWI) magnetic resonance imaging (MRI) in patients with acute bacterial rhinosinusitis Diffusion restriction in culture-positive acute sinusitis (diffusion-weighted image on the left and apparent diffusion coefficient map on the right, arrows point to restricted diffusion). Patient 1, 59-year-old female, status post resection of left neck liposarcoma. MRI is performed for follow-up, which demonstrates a central area of restricted diffusion within the left maxillary sinus (arrows). A culture of the sinus revealed beta-lactamase positive Bacteroides fragilis. Patient 2, 19-year-old male, presents with headache; an MRI shows a peripheral area of restricted diffusion within the right sphenoid sinus (arrows). A culture of the sinus revealed coagulase-negative Staphylococcus. Patient 3, 8-year-old boy, presents with longstanding headaches, an MRI performed shows severe mucosal thickening of the left frontal sinus with corresponding restricted diffusion. Culture of the sinus revealed gram-positive Streptococcus. Patient 4, 52-year-old male, undergoing trans-sphenoidal resection of a pituitary macroadenoma. MRI shows severe mucosal thickening of the right sphenoid sinus with corresponding restricted diffusion; this was cultured during surgery and showed Pseudomonas aeruginosa.

Based on the restricted diffusion in the sinuses, and despite the fact that the MRI was not performed because of sinus disease, the patients all had sinus cultures obtained and performed by the otolaryngology service. The following organisms were cultured: beta-lactamase positive Bacteroides fragilis, coagulase-negative Staphylococcus, gram-positive Streptococcus, and Pseudomonas aeruginosa. The key feature in all of these cases is that each had restricted diffusion within the sinus that was cultured, and found to be positive for bacteria (Figure 1).

## Discussion

To date, the diagnosis of acute rhinosinusitis has been a clinical dilemma despite recommendations from several specialty societies, including the Centers for Disease Control [6]. This has resulted in overuse of CT scanning, with unnecessary radiation exposure, and without definitive diagnosis given lack of defined CT imaging findings for acute bacterial rhinosinusitis. It has also lead to frequent prescribing of oral antibiotics for presumed acute bacterial rhinosinusitis despite the fact that most patients present with sinusitis secondary to viral etiologies. This overprescribing has contributed to the development of multi-drug resistant organisms. Over-prescribing oral antibiotics for non-proven sinusitis may be due to concern for potential complications from untreated acute rhinosinusitis, and decreased patient satisfaction if no medication is prescribed.

From a radiology standpoint, no clear imaging findings exist on CT for the diagnosis of acute bacterial rhinosinusitis, and therefore, diagnosis on imaging has always been controversial. The presence of a fluid-level is one of the more common findings suggested for acute rhinosinusitis, but can also be seen in the setting of viral and allergic sinusitis. In addition, air-fluid levels can be seen in chronic bed-ridden and trauma patients. Other findings that have been suggested in the literature include total filling/opacification of a sinus and mucosal thickening greater than 3 mm. However, none showed a correlation with positive bacterial cultures [7,8]. Studies have also shown that mucosal thickening can be a completely incidental finding on imaging [9]. For these reasons, it is clear that CT is not reliable for the diagnosis of acute bacterial rhinosinusitis, exposing patients to unnecessary radiation.

To the best of our knowledge, no study has been done to show that the presence of restricted diffusion within fluid or mucosal thickening in a sinus suggests the diagnosis of acute bacterial rhinosinusitis. One study suggests that lower apparent diffusion coefficient values are associated with mucosal thickening rather than other inflammatory lesions such as mucous retention cysts and air-fluid levels with homogeneous or heterogeneous T2-signal intensity [10]. No correlation with pathology was made on this study. All of the patients presented in this report had restricted diffusion within the sinus with confirmed positive bacterial cultures, consistent with acute bacterial rhinosinusitis. Perhaps a cutoff value for the apparent diffusion coefficient may be more useful to the radiologist in suggesting the diagnosis of acute sinusitis. We found that the signal on T2-weighted images in the area of restricted diffusion was variable. An associated fluid level within the sinus was also variable.

## Conclusions

The presence of restricted diffusion within a sinus may be a potential biomarker for acute bacterial sinusitis. A prospective study would be necessary in patients who clinically present with acute sinusitis. Techniques will need to eventually be optimized to address overutilization and cost issues. The sensitivity and specificity of MRI findings could be correlated with endoscopic and bacterial culture. It is conceivable that the presence of restricted diffusion in addition to other imaging features may offer greater sensitivity for the diagnosis. If DWI proves sensitive and specific, a simple scan of the sinuses with MRI could transform the practice of diagnosing acute bacterial rhinosinusitis, preventing unnecessary radiation to the patient and the overuse of antibiotics.
